# Menstrual cups and cash transfer to reduce sexual and reproductive harm and school dropout in adolescent schoolgirls in western Kenya: a cluster randomised controlled trial

**DOI:** 10.1016/j.eclinm.2023.102261

**Published:** 2023-10-10

**Authors:** Garazi Zulaika, Elizabeth Nyothach, Anna Maria van Eijk, Duolao Wang, Valarie Opollo, David Obor, Linda Mason, Tao Chen, Emily Kerubo, Boaz Oyaro, Alex Mwaki, Alie Eleveld, Isaac Ngere, Eunice Fwaya, Feiko O. ter Kuile, Daniel Kwaro, Penelope A. Phillips-Howard

**Affiliations:** a Department of Clinical Sciences, Liverpool School of Tropical Medicine (LSTM), Liverpool, UK; b Centre for Global Health Research, Kenya Medical Research Institute (KEMRI), Kisumu, Kenya; c Safe Water and AIDS Project (SWAP), Kisumu, Kenya; d Washington State University Global Health Program, Nairobi, Kenya; e Ministry of Health, Siaya County, Kenya

**Keywords:** Adolescent girls, HIV, HSV-2, Cash transfers, Menstrual cups, Kenya

## Abstract

**Background:**

High rates of sexual and reproductive health (SRH) harms and interrupted schooling are global challenges for adolescent girls, requiring effective interventions. We assessed the impact of menstrual cups (MCs) or cash transfers conditioned on school attendance (CCTs), or both, on SRH and schooling outcomes in western Kenya.

**Methods:**

In this cluster-randomised Cups or Cash for Girls (CCG) trial, adolescent girls in Forms two and three at 96 secondary schools in Siaya County (western Kenya) were randomised to receive either CCT, MC, combined CCT and MC, or control (1:1:1:1) for an average of 30 months. The CCT intervention comprised 1500KES (US$15 in 2016) via a cash card each school trimester. All four treatment groups received puberty and hygiene training. Assenting girls with parent or guardian consent who were post-menarche, not pregnant, area residents, not boarding, and had no disabilities precluding participation were eligible. Socio-behavioural risk factors and incidence of HIV and herpes simplex virus type 2 (HSV-2) were measured annually. School retainment and adverse events were monitored throughout. The primary outcome comprised a composite of incident HIV, HSV-2 and/or all-cause school dropout by school exit examination. The primary analysis was by intention-to-treat (ITT) using generalised linear mixed models, controlling for *a priori* selected baseline covariates. The trial is registered with ClinicalTrials.gov, NCT03051789.

**Findings:**

Between February 28, 2017 and June 30, 2021, 4137 girls (median age 17.1 [interquartile range (IQR): 16.3–18.0]) were enrolled and followed annually until completion of secondary school (median 2.5 years [IQR: 2.4–2.7]); 4106 (99.3%) contributed to the ITT analysis. No differences in the primary composite outcome between intervention and control groups were seen (MC: 18.2%, CCT: 22.1%, combined: 22.1%, control: 19.6%; adjusted risk ratio [aRR]: 0.97, 95% confidence interval 0.76–1.24; 1.14, 0.90–1.45; and 1.13, 0.90–1.43, respectively). Incident HSV-2 occurred in 8.6%, 13.3%, 14.8%, and 12% of the MC, CCT, combined and control groups, respectively (MC: RR: 0.67, 0.47–0.95, p = 0.027; aRR: 0.71, 0.50–1.01, p = 0.057; CCT: aRR: 1.02, 0.73–1.41, p = 0.92; combined aRR: 1.16, 0.85–2.58, p = 0.36). Incident HIV was low (MC: 1.2%, CCT: 1.5%, combined: 1.0%, and control: 1.4%; aRR: 0.88, 0.38–2.05, p = 0.77, aRR: 1.16, 0.51–2.62, p = 0.72, aRR: 0.80, 0.33–1.94, p = 0.62, respectively). No intervention decreased school dropout (MC: 11.2%, CCT: 12.4%, combined: 10.9%, control: 10.5%; aRR: 1.16, 0.86–1.57; 1.23, 0.91–1.65; and 1.06, 0.78–1.44, respectively). No related serious adverse events were seen.

**Interpretation:**

MCs, CCTs, or both, did not protect schoolgirls against a composite of deleterious harms. MCs appear protective against HSV-2. Studies of longer follow-up duration with objective measures of health impact are needed in this population.

**Funding:**

10.13039/501100000276Department of Health and Social Care, the 10.13039/501100020171Foreign, Commonwealth & Development Office, the 10.13039/501100000265Medical Research Council and 10.13039/100004440Wellcome.

**Research in context**

**Evidence before this study**

Poor menstrual health is widespread in low- and middle-income countries (LMIC), impacting girls’ health, wellbeing, and school engagement. However, the impact of menstrual interventions on objectively measured sexual and reproductive harms and schooling remain largely untested. Our pilot study in primary schoolgirls found that menstrual cups reduced rates of sexually transmitted infections (STI) and bacterial vaginosis. Girls reported engaging in transactional sex to obtain basic necessities, including sanitary pads, and identified cash needs as a primary driver for risky sexual behaviours. Cash transfers have been trialled among adolescents as potential interventions for HIV prevention. In Malawi, early results among adolescent girls showed conditional or unconditional cash transfers were associated with lower HIV and HSV-2 prevalence, risky sexual behaviours, and school dropout. As our trial commenced, two large studies were underway in South Africa and Kenya examining the effect of cash transfers and school support on HIV risk; no trials were in progress studying the menstrual cup on adolescent girls sexual and reproductive harms and/or schooling.

**Added value of this study**

Our cluster randomised controlled trial adds to a limited body of evidence on the effect of menstrual cups and/or conditional cash transfers on girls’ sexual and reproductive health and schooling outcomes. To our knowledge, it is the first large-scale trial testing the impact of a menstrual intervention on HIV and herpes simplex virus type 2 (HSV-2) incidence and/or all-cause school dropout. We found no evidence that the provision of menstrual cups protected against a combined outcome of incident HIV, HSV-2, and/or school dropout. However, menstrual cups appeared protective against HSV-2 acquisition, which is in alignment with emerging evidence showing cups do not disrupt the composition of the vaginal microbiome and reduce the incidence of bacterial vaginosis and STIs. Our results suggest that the menstrual cup, with adequate training and familiarisation, is a menstrual solution that may improve adolescent girls’ reproductive health in LMIC. We show no effect of conditional cash transfers on HIV or HSV-2 incidence, echoing findings from two other large trials testing similar structural interventions in adolescent girls in South Africa and Kenya.

**Implications of all the available evidence**

Interventions to reduce HIV and STI transmission, adolescent pregnancy, and incomplete schooling in this population are warranted. Menstrual cups are an acceptable and long-lasting solution to manage menstruation and may also protect girls’ reproductive health. School dropout remains intractable, requiring further intervention research. Longer term longitudinal evaluations of interventions targeting girls’ sexual and reproductive health as they transition into adulthood are needed.

## Introduction

Adolescent girls are uniquely susceptible to sexual and reproductive health (SRH) harms. This age group confronts many pubertal and environmental challenges, including incomplete biological development, lack of knowledge on menstruation and puberty, social pressures around sexual initiation, harmful gender norms, and entry into the workforce. In many places, including Kenya, where poverty and gender-based violence are pervasive, many girls face sexual abuse, have limited agency to navigate sexual encounters safely, or may resort to transactional sex to obtain basic necessities.^1^^,^^2^ These developments leave girls vulnerable to sexually transmitted infections (STIs), including HIV and herpes simplex virus type 2 (HSV-2), and to early pregnancy, with lasting ramifications across the life course.^3^

Consequently, adolescent girls face a disproportionate disease burden. It is estimated that in sub-Saharan Africa, girls are twice as likely as boys and young men to acquire HIV. While they comprised 10% of the population in 2020, they accounted for a quarter of all HIV infections in the region.^4^ In Kenya, the HIV prevalence in 2018 was 2.6% in females aged 15–24 and 1.3% in males.^5^ Adolescents are at the epicentre of the country’s HIV epidemic, with 42% of all new infections in 2020 occurring in this age group.^6^ HIV shares risk factors and structural determinants with other STIs, including HSV-2, which girls typically acquire soon after sexual debut.^7^ HSV-2 seroprevalence in Kenya, at 39.9% (95% confidence interval (95% CI): 34.3–45.9), is five times that of all of Europe.^7^ By age 17, 17% of schoolgirls are seropositive for HSV-2 in western Kenya.^8^ Moreover, three in five girls become pregnant before age 19, often accompanied by severe maternal health risks and interrupted schooling.^9^ These figures highlight the heightened vulnerability of this population to life-long harms, and underscore the importance of prevention efforts at this key juncture.

Prevention efforts attempt to disrupt the behavioural and structural drivers associated with these harms. Behavioural interventions aiming to prevent adolescent HIV transmission, delay sexual debut, and decrease the number of sexual partners and risky behaviours have had limited success in Africa.^10^^,^^11^ Cash transfer (CT) programmes have been trialled among adolescents as potential structural interventions for HIV prevention, with mixed results. Such interventions are believed to improve girls’ economic purchasing power, allowing them to obtain items without relying on sex or older partners. Two recent trials saw no direct intervention effect on HIV or HSV-2 incidence.^12^, ^13^ A trial in school-going girls in Malawi detected a lower prevalence of HIV and HSV-2 among those receiving CT, although baseline seroprevalence of HIV and HSV-2 was not captured or accounted for in the analysis.^14^ CT has shown more positive results on impacting girls’ risky sexual behaviours, with studies finding direct improvements in the age at sexual debut, number of sexual partners, delayed childbearing, and incidence of transactional sex, age-disparate sex, and unprotected sex.^12^^,^^14^^,^^15^

Others have tested CT and its impacts on schooling, a key pathway to improving adolescents’ SRH.^14^^,^^16^ Schools act as safe spaces that protect girls from SRH harms, especially against adolescent pregnancy.^17^ Menstruation and the lack of appropriate hygiene materials have been reported in several qualitative studies as barriers to girls’ schooling.^1^^,^^18^ Accordingly, research has started investigating menstrual solutions for schoolgirls to determine if they can positively impact education and reduce SRH harms. Studies have largely tried to measure the impact on school attendance, but girls’ reports of menstruation-related absence have proven challenging to capture and quantify, underscoring the need to study other related outcomes.^19^ Our previous pilot study in western Kenya was unable to reliably measure impacts on school absence or dropout, but found that providing menstrual products to schoolgirls, particularly reusable menstrual cups (MCs), reduced STIs and reproductive tract infections,^20^ further encouraging calls for larger robust trials on menstrual solutions.^21^ We present the findings from a cluster randomised trial in western Kenya evaluating the effect of MCs and conditional cash transfers (CCTs) on schoolgirls’ risk of HIV, HSV-2 and school dropout.

## Methods

### Study design and participants

The Cups or Cash for Girls (CCG) Trial was a four-group cluster randomised controlled trial which took place in 96 secondary schools in Siaya County, western Kenya, as described elsewhere.^22^ Schools were block randomised into four treatment arms (1:1:1:1): (a) girls in MC allocated schools received one menstrual cup and soap for hand-washing (“MC”), (b) girls in cash schools received KES 1500 per term (∼US$15), conditional on 80% attendance in the prior term (“CCT”), (c) girls in combined intervention schools (“combined”) received both MC and CCT, and (d) girls in the usual practice arm (“control”) received soap for handwashing and, at trial close, one MC each. The study area is characterised by poverty, disproportionate numbers of school dropouts amongst girls, and high SRH risks, including HIV and HSV-2, among adolescents.^8^

Secondary day schools with head teacher approval to participate that were not boys only schools, exclusively boarding, or for children with special needs were eligible. Girls were eligible if they were day-scholars (non-boarders), in select class years, had a history of three or more menses, no visible or declared pregnancy, no disability precluding participation, and resided in the study area.

### Ethics

The study was approved by the Kenya Medical Research Institute (protocol #3215) and the Liverpool School of Tropical Medicine (protocol #15-005). Each girl and her parent or guardian provided written assent and informed consent to participate. An independent Trial Steering Committee provided scientific guidance and monitored the progress of the trial, adherence to standards, and consideration of participants’ rights and safety. The trial’s Data Monitoring and Ethics Committee (DMEC) assessed and reviewed the trial data to ensure participants were not subjected to any excess risk, monitored intervention roll-out and adverse events, and provided scientific guidance. The DMEC Chair and independent statistician approved the statistical analysis plan (SAP) prior to the unblinding of trial data. Both committees met annually throughout the trial.

### Randomisation and masking

A census of all secondary female-only and coeducational day schools built the study area sampling frame. Block randomised groupings of four schools (“quads”) based on location and size were prepared by the trial statistician. School recruitment ceremonies in each sub-county invited head teachers per quad to select one of four unidentified coloured balls. After all quads completed this selection, a Ministry of Education representative opened a sealed envelope and disclosed the colour-to-intervention allocation. Participants were assigned to treatment groups based on their school’s random assignment. While blinding of study participants and field staff was not possible, girls were not informed of the allocation of other schools, and the laboratory staff, study investigators, and statisticians were blinded to participant allocation until all data were locked.

### Procedures

Eligible girls were familiarised with Android tablets and asked to complete a self-administered sociodemographic and behavioural survey in English or Dholuo annually. Girls’ height, weight, and waist measurements were measured by trained field staff. Girls individually received HIV counselling before providing a blood specimen for HIV and HSV-2 testing. Test results were returned to girls via their selected health facilities. Nurses visited schools to inform all participants that their results were ready for collection at their discretion, in order to maintain confidentiality and provide post-test counselling and linkage to care. Nurses liaised with health facilities, schools, and households to monitor adverse events among participants.

At the school level, data were routinely collected on adverse events, pregnancies, and school absence via focal point teachers and through school registers, collected termly. Attendance rates were calculated from school registers for the CCT intervention, and girls who routinely did not attend class were visited at home to document the reason for absence (including pregnancy), and to confirm whether they had dropped out or transferred to a new school. School water, sanitation, and hygiene (WASH) facilities were assessed annually, during an unscheduled site visit, to understand schoolgirls’ access to toilets and water for handwashing and to capture if any other programmes leading to potential trial contamination were ongoing.

### Interventions

The CCT intervention comprised 1500KES (∼US$15 in 2016) each school trimester. CCT participants received a chargeable Equity Bank cash card to withdraw money at decentralised bank agents. Money was sent directly to the participant’s card each school term and was conditional on attending 80% of days in the prior term. Girls had to provide a birth certificate and a parent/guardian ID to receive a cash card. In Malawi, Baird and colleagues identified US$5 per month as a sufficient amount to produce intervention effects.^14^ We selected this amount to provide girls some agency over their personal financial expenditures. All CCT participants received financial literacy training, including information on safety; bank agents familiarised girls with banking and card use.

The MC intervention comprised one *Mooncup*® (FDA #3009117944). The cup was replaced if it was reported lost, stolen, or damaged. The MC is a medical-grade silicone-based receptacle that is inserted into the vagina to collect menstrual blood and can last ten years.^23^ MCs are used internationally and are considered safe, cost-effective, and environmentally friendly for menstruation management.^23^ All girls receiving MCs were sensitised and trained on appropriate cup use and safekeeping; study nurses retrained girls requesting additional guidance.

Girls in all four treatment groups received puberty and hygiene training post enrolment.

### Outcomes

The primary outcome was a composite of SRH and education harms comprising incident HIV, HSV-2, and all-cause school dropout. The key secondary outcomes included the individual composite components (school dropout, HIV and HSV-2) and adolescent pregnancy. The primary safety endpoints were the incidence of toxic shock syndrome among cup using girls, and violence associated with cash use. All behavioural and biological outcome data were collected at annual timepoints; monitoring data were collected routinely throughout follow-up. No outcome data were imputed for analysis and all outcomes assessed were prespecified in the study protocol.

### Statistical analysis

96 school clusters (24 per arm) with an average of 41.25 girls per school were required to obtain 90% power to detect a 25% reduction in the primary endpoint from 39.3% in the control arm to 29.5% in any intervention arm (alpha = 0.0167 to allow for three primary comparisons) (Appendix p 3).

The primary endpoint was based on the intention-to-treat (ITT) population, defined as the population of all enrolled participants. Generalised linear mixed models (GLMMs) were fitted with treatment as a fixed effect and quad and school cluster as random effects to allow for the hierarchical structure of the study design. We calculated unadjusted risk ratios (RR) and their corresponding 95% CI by fitting the GLMMs to individual follow-up data with treatment status, and adjusted risk ratios (aRR) by controlling for participant age, baseline HIV and HSV-2 status, household SES, sub-county, school population size, and school baseline WASH conditions in SAS v9.3 and in STATA v17.0. For continuous outcomes, the models were fitted using a Gaussian distribution and identity link function; for binary and categorical data log-binomial models were employed. If the log-binomial models did not converge, Poisson distributions were used keeping only school as a random effect. For time-to-event outcomes, cox regression models were used to generate hazard ratios (HR) and 95% CI. The study was designed as a 4-way parallel trial because interactions between interventions (CCTs and MCs) were considered plausible. However, we also conducted a secondary 2 × 2 factorial “at the margins” analysis to estimate the pooled effect of MC vs no MC and CCT vs no CCT on the primary composite outcome and assessed for interactions.

Subgroup analyses of the primary outcome were conducted by age group, study location, socioeconomic profile, and sexual debut history at baseline. A per-protocol (PP) secondary analysis of the primary outcome included only girls who reported using their allocated treatment (i.e., reported using MC or withdrew money from their cash-card). Sensitivity analyses of the PP population were conducted to determine whether serious bias was present in the PP analysis for the effect of MC on dropout, due to the high likelihood for differential selection bias. While data on cash withdrawal in the cash and combined groups were available continuously throughout the study, data on cup use were only collected at the mid-study and end-of-study surveys. Thus, girls that dropped out before the mid-study survey had no data on cup use and were excluded from the PP analysis. To check for selection bias, we (1) modelled all MC and combined group girls who had dropped out before a second survey as cup “users”; (2) restricted the time under follow-up to the period between mid-survey when use data for cups was reported and end-of-study (avg. follow-up to mid-study 1.1 years); and (3) ran Kaplan–Meier survival estimates for dropout for this restricted period. All primary and secondary analysis methods were prespecified in the trial’s SAP; the sensitivity analyses checking for bias in the PP estimates were conducted post-hoc.

Additional methods on sociodemographic and WASH measures are provided in the Supplementary Material (Appendix p 3). The trial was registered with ClinicalTrials.gov, NCT03051789.

### Role of funding source

The funder had no role in the study design, data collection and analysis, interpretation of the trial findings, decision to publish, or preparation of the manuscript.

## Results

**Fig. 1:**
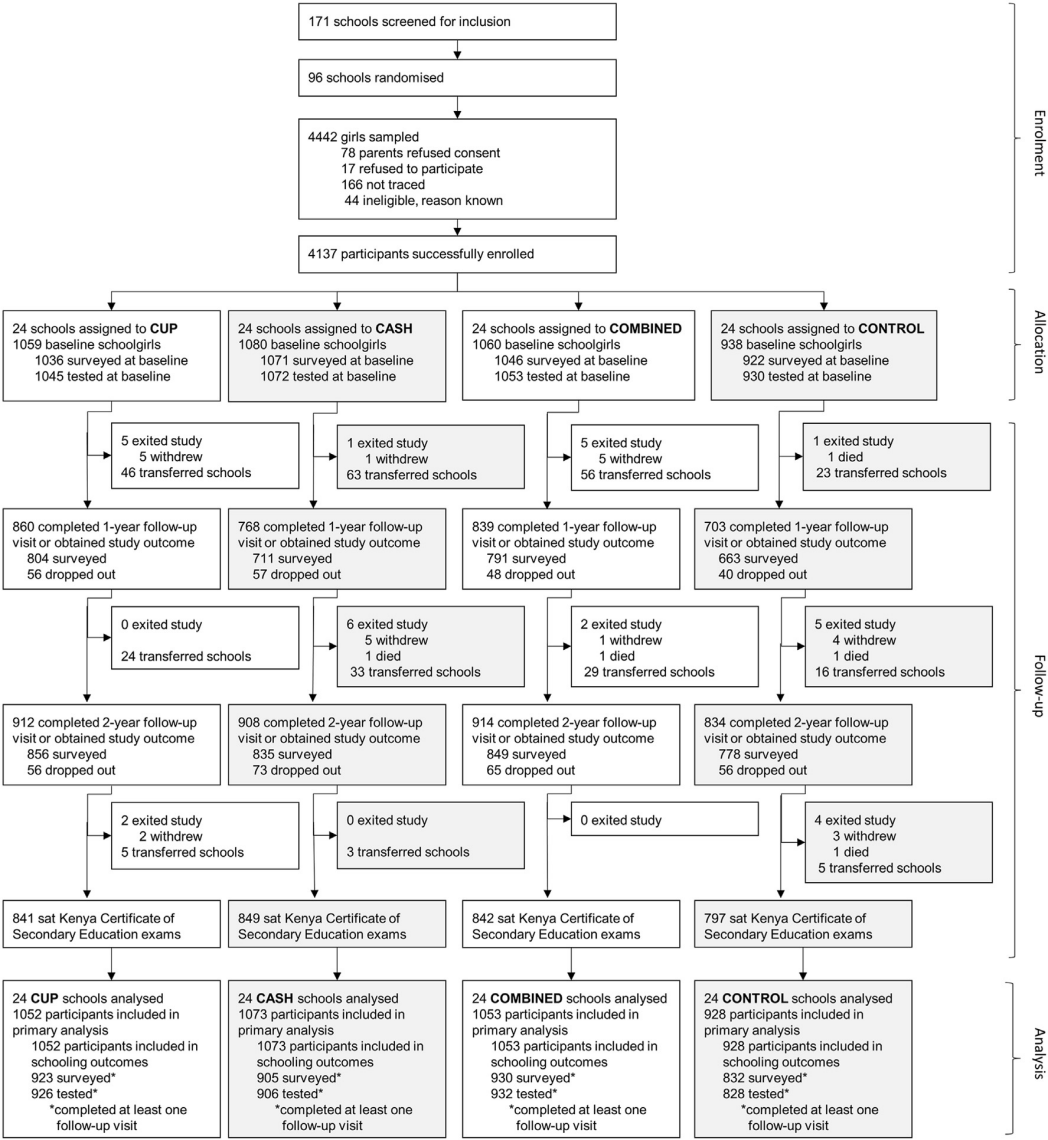
Trial profile. Definitions: “surveyed” refers to girls completing the sociodemographic and behavioural questionnaire; “tested” refers to participants having available laboratory confirmed biomarker data; “dropped out” refers to obtaining the outcome “school dropout.”

Between February 28, 2017 and July 6, 2018, 96 schools were enrolled (Fig. 1), including 4137 girls, of whom 4106 completed the trial and 31 were lost to follow-up due to withdrawal from the study (n = 27) or death (n = 4) by end of follow-up (June 30, 2021). Baseline HIV and HSV-2 status was available for all girls; incident HIV or HSV-2 infection could not be determined in 514 (12.5%). The number of participants lost to follow-up and those without incident HIV and HSV-2 data were similar between intervention groups (Fig. 1).

The median age at enrolment was 17.1 years (IQR 16.3–18.0 years). Baseline characteristics in the four arms were generally well balanced (Table 1), with the exception that a larger proportion of girls in the CCT and combined groups had a history of pregnancy at baseline, and a greater number of girls in the CCT group were in larger schools — on average larger schools had poorer WASH conditions than smaller schools (Table 1).

**Table 1:** Baseline sample characteristics by intervention arm among all enrolled into the Cups or Cash for Girls Trial (n = 4137).

	Total population	CUP group	CASH group	COMBINED group	CONTROL group
	(N = 4137)	(N = 1059)	(N = 1080)	(N = 1060)	(N = 938)
Age, median (interquartile range)	17.10 (16.3–18.0)	16.91 (16.1–17.8)	17.20 (16.4–18.1)	17.19 (16.3–18.1)	17.10 (16.4–17.9)
Age group ≤17 years	1965 (47.5%)	564 (53.3%)	484 (44.8%)	476 (44.9%)	441 (47.0%)
Socioeconomic status (lowest two quintiles)	1758 (42.7%)	401 (38.0%)	498 (46.3%)	483 (45.8%)	376 (40.4%)
Married	265 (6.5%)	73 (7.0%)	66 (6.2%)	58 (5.5%)	68 (7.4%)
Orphaned	137 (3.3%)	34 (3.2%)	41 (3.8%)	37 (3.5%)	25 (2.7%)
HIV positive	75 (1.8%)	13 (1.2%)	20 (1.9%)	23 (2.2%)	19 (2.0%)
Herpes simplex virus type 2 positive	701 (17.3%)	171 (16.5%)	193 (18.3%)	163 (15.6%)	174 (18.9%)
Early menarche <13 years	236 (5.7%)	58 (5.5%)	53 (4.9%)	66 (6.2%)	59 (6.3%)
Sexually active	1124 (27.6%)	292 (28.2%)	305 (28.5%)	277 (26.5%)	250 (27.1%)
Prior pregnancy	141 (12.2%)	27 (9.0%)	47 (15.2%)	41 (14.2%)	26 (10.1%)
School size >46 girls	2034 (49.2%)	499 (47.1%)	634 (58.7%)	508 (47.9%)	393 (41.9%)
Water, sanitation, and hygiene school score <1	2351 (56.8%)	513 (48.4%)	797 (73.8%)	573 (54.1%)	468 (49.9%)
Sub-county	–	–	–	–	–
Gem	981 (23.7%)	260 (24.6%)	215 (19.9%)	323 (30.5%)	183 (19.5%)
Rarieda	910 (22.0%)	216 (20.4%)	240 (22.2%)	230 (21.7%)	224 (23.9%)
Siaya	828 (20.0%)	213 (20.1%)	215 (19.9%)	172 (16.2%)	228 (24.3%)
Ugenya	793 (19.2%)	209 (19.7%)	220 (20.4%)	193 (18.2%)	171 (18.2%)
Ugunja	625 (15.1%)	161 (15.2%)	190 (17.6%)	142 (13.4%)	132 (14.1%)

### Primary composite and key individual outcomes

The primary outcome of HIV and HSV-2 incident infection and/or all-cause school dropout was 20.5% across all arms. There were 44 incident HIV infections and 364 HSV-2 infections during the trial (cumulative risk 1.25% and 12.2%, respectively). The prevalence of HIV and HSV-2 at enrolment was 1.8% and 17.3% and reached 2.9% and 25.8% by study end, respectively. Overall, 11.3% of girls dropped out of school, of whom 54.3% dropped out due to pregnancy and 14.7% due to marriage (Appendix p 4).

**Fig. 2:**
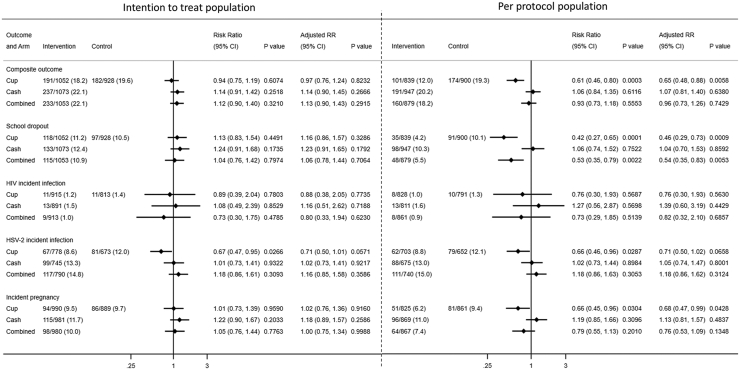
Primary composite outcome and individual outcomes of incident HIV, herpes simplex virus type 2, school dropout and pregnancy. Abbreviations: HSV-2, herpes simplex virus type 2; RR, risk ratio; CI, confidence interval.

No significant difference in the primary outcome was found between treatment groups in the ITT population: MC: 18.2%, CT: 22.1%, combined: 22.1%, and control: 19.6% (aRR vs control: MC: 0.97, 95% CI 0.76–1.24, p = 0.82; CT: 1.14, 0.90–1.45, p = 0.27; and combined: 1.13, 0.90–1.43, p = 0.29, Fig. 2, intra-class correlation coefficient = 0.0743). Analysis of the individual components of the primary outcome showed that there was a 33% reduction in HSV-2 acquisition in the MC group (8.6%) against control (12.0%) before adjustment (RR: 0.67, 0.47–0.95, p = 0.027) and 29% after adjustment (aRR: 0.71, 0.50–1.01, p = 0.057). No reduction was seen in the combined or CCT groups (combined: 14.8%, aRR: 1.16, 0.85–2.58, p = 0.36; CCT: 13.3%, aRR: 1.02, 0.73–1.41, p = 0.92). MCs were associated with a lower but statistically non-significant difference in HIV incidence relative to the control group (1.2% vs 1.4%, aRR = 0.88, 0.38–2.05, p = 0.77), as was the combined intervention (1.0%, 0.80, 0.22–1.94, p = 0.62), but not CCT group (1.5%, aRR: 1.16, 0.51–2.62, p = 0.72). No intervention was associated with reductions in school dropout nor with adolescent pregnancy (Fig. 2, ITT population).

83.0% of girls in the combined arm used both interventions; cup use was reported by 79.3% of girls in the cup arm (81.2% across both groups) and 87.8% of CCT group girls withdrew cash at least once (85.5% across both), resulting in a PP population of 3579 of 4137 girls (86.5%). The incidence of the primary composite endpoint in this PP population was 12.0% in the MC group, 20.2% in the CCT group, 18.2% in the combined group, and 19.3% in the control group. Relative to the control group, there was no reduction in the primary composite outcome in the CCT (aRR: 1.07, 0.81–1.40, p = 0.63) or combined groups (aRR: 0.96, 0.73–1.26, p = 0.74) (Fig. 2, PP population). However, there was a 35% reduction in the primary outcome in the MC group compared with controls (aRR: 0.65, 0.48–0.88, p < 0.0058, Fig. 2, PP population), with a corresponding 54% reduction in school dropout (aRR = 0.46, 0.29–0.73, p = 0.0009) and pregnancy during the trial (aRR: 0.68, 0.47–0.99, p < 0.043), and smaller non-significant reductions in HSV-2 (aRR: 0.71, 0.50–1.02, p < 0.066) and HIV incidence (aRR = 0.76, 0.30–1.93, p = 0.56). The combined intervention was also associated with lower school dropout (aRR = 0.54, 0.35–0.83, p = 0.0053), but not HSV-2 (aRR = 1.18, 0.86–1.62, p = 0.31), HIV (aRR = 0.82, 0.32–2.10, p = 0.69), or incident pregnancy (aRR = 0.76, 0.53–1.09, p = 0.13). No reductions in any component of the primary outcome or pregnancy were seen in the CCT group PP analysis.

### Sensitivity analysis

In the first PP sensitivity analysis, where early dropouts were re-classified as “cup users,” all effects seen in the main PP analysis on dropout and pregnancy disappeared (Appendix p 5). The second sensitivity analysis with restricted follow-up between mid-study survey when cup use was reported and end-of-study echoed the main PP results, showing a large reduction in the composite outcome in the MC group but not for the combined group or CCT group (MC: aRR: 0.61, 0.47, 0.80, p = 0.0002, combined: aRR: 0.88, 0.70–1.10, p = 0.26, CCT: 1.09, 0.87–1.37, p = 0.44, Appendix p 5). The time-to-event sensitivity check for the individual outcome school dropout similarly showed a reduction in dropout for the MC group but not for the combined group (MC: HR: 0.56, 0.31, 1.01, p = 0.055, combined: HR: 0.89, 0.52–1.54, p = 0.69, Appendix p 9). These analyses suggest bias may have been introduced into the PP results by early dropouts with missing information on intervention use.

### Subgroup analysis

Subgroup analyses for differences in the relative risk of the composite outcome were run for age group, socioeconomic status, school size, school WASH conditions, baseline HIV or HSV-2 seropositivity, sexual activity at baseline, and experiencing the COVID-19 school closures; no meaningful differences were noted between subgroups (Appendix p 8).

### Safety outcomes

No instances of toxic shock syndrome were observed during the trial. Ten serious adverse events were reported, and 25 adverse events occurred throughout the study. Five girls died during the study with causalities unrelated to trial interventions (Table 2). Nine adverse events were related to girls’ intervention use; none of these required hospital admission.

**Table 2:** Safety outcomes (Safety Population).

	Total	Cups	Cash	Combined	Controls
	(N = 4137)	(N = 1059)	(N = 1080)	(N = 1060)	(N = 938)
All serious adverse events^a^	(n = 10)	(n = 2)	(n = 3)	(n = 1)	(n = 4)
Primary safety outcome [Toxic shock syndrome]	0	0	0	0	0
Deaths^b^	5	0	1	1	3
Hospital admissions without death^c^	5	2	2	0	1
Adverse events related to intervention	(n = 9)	(n = 3)	(n = 0)	(n = 6)	(n = 0)
Cup retention	1	1	0	0	0
Vaginal (cup) discomfort	5	2	0	3	0
Domestic violence	3	0	0	3	0
Adverse events not related to intervention	(n = 16)	(n = 4)	(n = 4)	(n = 5)	(n = 3)
Pain during intercourse	1	1	0	0	0
Vaginal discomfort	9	1	1	4	3
Domestic violence	1	0	1	0	0
Pregnancy complications	1	0	1	0	0
Injuries–wounds and fractures	1	1	0	0	0
Respiratory infections and asthma	1	1	0	0	0
Other	2	0	1	1	0

a Serious adverse events are any adverse event or adverse reaction that results in death, is life-threatening, requires hospitalisation or prolongation of existing hospitalisation, results in persistent or significant disability or incapacity.

b Causes of death: road traffic injury, pregnancy complication, rheumatic heart disease, organ failure post-infection, and advanced duodenal ulcers.

c Causes of hospital admissions not resulting in death: malaria, childbirth, anal swelling, septic wound, and fractures related to a pedestrian road accident.

## Discussion

This is the first cluster randomised controlled trial studying the effects of MC and CCT interventions on adolescent schoolgirls’ SRH and schooling. While neither intervention, alone or in combination, impacted the primary composite outcome of incident HIV, HSV-2, and/or all-cause school dropout, a borderline statistically significant, but clinically relevant 29% (95% CI −1% to 50%) lower incidence of HSV-2 acquisition was observed among MC group girls.

MCs’ protective effect on HSV-2 acquisition has plausible biological and behavioural mechanisms of action. MCs have been shown to preserve the *Lactobacillus-*dominant vaginal bacterial environment.^24^ A *Lactobacillus*-dominant vaginal microbiome, in particular one that is *Lactobacillus crispatus* dominant, is protective of bacterial vaginosis (BV) and STIs, including HSV-2.^25^^,^^26^ Evidence is building that menstrual materials, including disposable and reusable pads, are associated with a non-optimal vaginal microbiome, BV, and other reproductive tract infections.^20^ Our earlier study found that MCs reduced BV prevalence in schoolgirls both relative to controls and to girls who received disposable pads.^20^ MCs may prevent acquisition of HSV-2 through promotion and/or maintenance of the *Lactobacillus*-dominant vaginal microbiome. In a nested sub-study measuring cups’ effects on the vaginal microbiome of 436 participants in the current trial, we showed that MCs were associated with a higher occurrence of *L. crispatus* dominated Community State Type 1 (CST-1) (odds ratio 1.37 [95% CI 1.06–1.75]) than girls in the control group, and higher relative abundance of *L. crispatus* (mean difference 3.95% [95% CI 1.92–5.99]) with a concomitant 24% decrease in the odds of BV (odds ratio 0.76 [95% CI 0.59–0.98]).^27^ Our study adds to the growing evidence on MCs’ beneficial impact on the reproductive tract, showing that downstream infections like HSV-2 can be prevented through certain menstrual interventions. And, while no direct effects on HIV incidence were seen during the trial, up to 15% of HIV infections are estimated to be attributed to BV as observed in meta-analysis,^25^ and HSV-2 is associated with a 5-fold increased risk of HIV,^28^ suggesting MCs may contribute to reduced HIV acquisition over time.

Studies have documented that MC uptake is not immediate, with girls requiring up to six months use for familiarisation.^23^^,^^29^ Reported MC use increased throughout follow-up (Appendix p 9), with more than four in five cup recipients reporting use during their last period and three in four girls reporting emptying their MC at school without issue by end of study. However, girls continued using other menstrual products in tandem with their MCs, with combined group girls reporting more pad use at end-of-study than at midline (Appendix p 9). These girls also reported using their CT to purchase sanitary pads (Appendix p 7), highlighting that girls opt to use multiple menstrual products per cycle if available. It is possible that higher pad accessibility through the CT in the combined group allowed girls to primarily use pads, limiting MC familiarisation and uptake and decreasing the MCs’ biological benefits. As seen in the pooled effects analysis (Appendix p 6), among MC using girls, MCs were strongly protective against the primary outcome but interacted with the CCT in the crude analysis (p_interaction_ = 0.048). While this interaction disappeared after adjustment, it suggests that CCT receipt was an effect modifier of the MC effect, and that MCs worked best when CCT was not also provided. The MC intervention requires training and continued support to ensure girls feel comfortable using the product. Yet, once familiarised, users report high levels of satisfaction in using the product to manage their menses. One recent systematic review found that MC users voiced decreased stress of staining and improved mobility when using the cup, and three in four participants reported wishing to continue using the product.^23^

MCs were not associated with reductions in school dropout in this study population. Previous studies have consistently found that girls’ self-report heightened absenteeism during their periods, citing menstrual shame, fear of leaking or odour, and poor WASH facilities at school as the primary causes.^1^^,^^30^ However, in our study the primary driver of dropout was pregnancy (Appendix p 4), indicating menstruation-related absence may not be a major attributor to school dropout. In this setting, adolescent pregnancy has been an intractable challenge, and is both a cause and a consequence of school dropout among secondary schoolgirls.^3^ One recent study by Kangwana and colleagues has suggested that earlier intervention during adolescence may generate protective factors against adolescent pregnancy, suggesting these might have spillover benefits on education and girls’ longer term health and economic outcomes.^15^ More work is required to identify interventions to help prevent early motherhood in these settings.

CCT was not associated with any reduction in school dropout. Our findings align with a recent large trial in South Africa that gave monetary CT directly to recipients and similarly found no effect on school dropout among secondary schoolgirls.^12^ Additionally, CT had no effect on HIV or HSV-2 incidence, in line with other CT intervention trials studying these biomarkers.^12^ Two studies reported improvements in HIV and HSV-2 prevalence; however, in both studies, no baseline measurements were taken, limiting comparability between groups.^14^^,^^16^ Recently, a multicomponent intervention study testing financial incentives and educational subsidies among adolescent girls in our study area also found no improvement in HIV or HSV-2 rates^11^; while another among younger adolescents in an informal urban area of Nairobi reported a modest reduction in HSV-2 rates among a subset of girls receiving CT, again suggesting earlier intervention may be of value.^15^ Qualitative interviews have often highlighted CT recipients’ wish for additional money, stating the CT is insufficient to cover needs. Furthermore, experts have argued that transactional sex is often not strictly for basic survival, but also to acquire affection or items girls desire and may become a mode for improved social status.^31^ Thus, while CT supporters have argued that a small amount of money can reduce risky sexual behaviours by providing for basic goods, it is unlikely that CT alone prevents all risky sexual behaviours.

It is plausible that the CT amount given in our trial was too low to yield any large impacts on objective measures of girls’ SRH and schooling. Most CT programmes have opted to channel CT to girls via their parents or guardians.^15^ Of those giving money to girls directly, most have also included a separate parent or guardian incentive.^12^^,^^14^ Our design was unique because we gave money directly to girls. We believed this would support their self-autonomy and improve their financial knowledge when paired with financial literacy training and bank familiarisation. However, one in seven girls reported their parents kept their cash cards, and one in two reported sharing their CT with family members (Appendix p. 7). Consequently, more attention needs to be given to household economic barriers when designing interventions to impact adolescent girls’ SRH and schooling.

Our CCT intervention design followed Baird and colleagues (2012) trial findings in Malawi.^14^ They tested variable transfer amounts given to both parents and participants and concluded that a minimum amount of US$5 a month was sufficient to see the effects on schooling.^32^ We used the suggested benchmark of US$5 per month for the months girls were in school (∼9 months). The resulting transfer per year effectively came to US$45 per year relative to Baird and colleagues’ US$60 per year, and included no parent incentive. Throughout the trial, inflation and cost of living steadily rose in Kenya, lowering the net household income effect relative to the Malawi study. Notably, including a parent incentive or larger CT amount could yield unsustainable scale-up costs for LMIC.

Our trial had some limitations. First, the COVID-19 pandemic occurred during the trial, leading to interrupted intervention provision for the months that schools were closed in Kenya and fieldwork was suspended. This disruption may have biased the real effects of each intervention towards the null. Second, all sexual activity measures were self-reported and likely reflect socially desirable responses that underreport sexual activity. Cup intervention use was also self-reported with no confirmed measurement. Third, the observed occurrence of the primary outcome was below the assumed parameters used in the sample size calculation, potentially underpowering the primary analysis. Moreover, this primary composite measure of school dropout, and incident HSV-2 and HIV infection included three outcomes that are not contemporaneous and may differ temporally on the causal pathway.^11^ School dropout is proximal, only occurring during schooling; however, measuring schooling’s impact on HSV-2 acquisition, and whether any improvements attenuate HIV risk, requires additional follow-up time for accurate measurement. Fourth, end-of-study surveys and biospecimen collection occurred prior to final school exams, resulting in shorter follow-up time for non-schooling outcomes, possibly reducing the intervention effect on HIV and HSV-2 incidence and explanatory SRH values. Additionally, there may be selection bias in our ITT analysis due to omitting individuals with missing HIV or HSV-2 outcome data. Fifth, meaningful causal inferences cannot be drawn from any PP analyses comparing only treatment users in an intervention arm to all participants in a control arm, as they violate the principles of randomisation and are subject to confounding. For example, our PP analysis that included only girls reporting using the intervention may have introduced substantial selection bias by not including girls who dropped out before completing a follow-up survey where MC use data were collected in the two MC arms (MC and combined groups). Meanwhile, early dropouts in the other arms were included, as MC use data were not applicable to these groups. Conversely, the alternative PP analysis that assumed these early dropouts in the MC arms were “MC users,” would possibly underestimate any intervention effects as some girls who exited early may not have used their MC.

Strengths of our study included the large sample size and a high rate of follow-up. We randomised at the school-level to avoid spillover effects of the intervention. Our study measured objective outcomes, with laboratory confirmed biomarker data at baseline and each follow-up round, and individually monitored dropout with confirmatory home visits for all participants across 2500 square kms. We evaluated a menstrual solution, which has received little attention in adolescent SRH intervention research, against CCT. Our schoolgirl population is generalisable to other impoverished rural LMIC settings.

In conclusion, overall, we found no evidence that providing MC and/or CCT reduces the composite of incident school dropout, HIV and/or HSV-2, or had any effect on dropout alone. However, MCs appeared protective against HSV-2 acquisition, adding evidence that MC use may protect the reproductive tract. MCs are a one-time, environmentally friendly and sustainable low-cost intervention.^23^ Our findings indicate that a menstrual product intervention, beyond improving girls’ dignity and comfort in managing their menses, may have beneficial effects on adolescent girls’ reproductive health and is worthy of consideration by policymakers as an intervention to support girls’ health and development. CCT interventions were ineffective as designed in this population and appear very context-sensitive and resource heavy, requiring more piloting and adaptation.

## Contributors

PPH, LM, FtK, and DW conceptualised the research question. PPH, FtK, GZ, DW, DK, LM, AMvE, EN, and TC designed the methodology. EN, GZ, DO, DK, AE, AM, IN, and EF conducted the investigation under the supervision of PPH and DK. The project was administered by PPH, EN, GZ, AE, AM, EF, IN, and LM. Laboratory resources were provided by VO, BO, and EK. AMvE, TC, and GZ accessed and verified the underlying data. DW conducted the formal analysis, which was validated by GZ, AMvE, and TC. GZ, PPH, AMvE, LM, DW, and FtK led data interpretation and wrote the original draft. All investigators had access to the data, contributed to the review and editing of the manuscript, and approved the final version.

## Data sharing statement

This study was conducted with approval from the Kenya Medical Research Institute (KEMRI) Scientific and Ethics Review Unit (SERU), which requires that de-identified data from any Kenya-based study be released only after receipt of written KEMRI SERU approval for additional analyses. In accordance, trial data will be available upon request, after obtaining written KEMRI SERU approval for the proposed analysis. Application forms and guidelines can be accessed at https://www.kemri.org/seru-overview or by contacting seru@kemri.org.

## Declaration of interests

We declare no competing interests.
