# Supplementary material for: Menstrual cups and cash transfer to reduce sexual and reproductive harm and school dropout in adolescent schoolgirls in western Kenya: a cluster randomised controlled trial
Source: eClinicalMedicine. 2023 Oct 10;65:102261. doi: 10.1016/j.eclinm.2023.102261 (PMC10582356; doi:10.1016/j.eclinm.2023.102261)
Supplement: Supplementary Materials [file mmc1.docx]

**Supplementary materials**

**Supplement to: Menstrual cups and cash transfer to reduce sexual and reproductive harm and school dropout in adolescent schoolgirls: a cluster randomised controlled trial in western Kenya**

Garazi Zulaika PhD^1§^, Elizabeth Nyothach MA^2^, Annemieke van Eijk PhD^1^, Duolao Wang PhD^1^, Valarie Opollo PhD^2^, David Obor MSc^2^, Linda Mason PhD^1^, Tao Chen PhD^1^, Emily Kerubo BSc^2^, Boaz Oyaro BSc^2^, Alex Mwaki BSc ^3^, Alie Eleveld MPH^3^, Isaac Ngere PhD^4^, Eunice Fwaya, Bpharm^6^, Feiko O. ter Kuile PhD^1^, Daniel Kwaro MPH^2^, Penelope A. Phillips-Howard, PhD^1§^

**Table of Contents**

[**Supplemental methods:** 3](#_Toc138690135)

[Scales 3](#_Toc138690136)

[Sample size re-estimation 3](#_Toc138690137)

[Other secondary outcomes 3](#_Toc138690138)

[**Deviations from the study protocol:** 3](#_Toc138690139)

[**Supplemental references:** 4](#_Toc138690140)

[**Supplemental tables:** 4](#_Toc138690141)

[Supplemental Table 1: Reasons for dropout (n=464; among total 4137 participants enrolled) 4](#_Toc138690142)

[Supplemental Table 2: Baseline characteristics in the per protocol population, overall and by arm 4](#_Toc138690143)

[Supplemental Table 3: Per protocol population sensitivity analysis of primary outcome and disaggregated components 5](#_Toc138690144)

[Supplemental Table 4: Per protocol population restricted follow-up analysis of primary outcome and disaggregated components 5](#_Toc138690145)

[Supplemental Table 5: At the margins pooled factorial analysis for the primary oucome 6](#_Toc138690146)

[Supplemental Table 6: Cash use characteristics among girls receiving cash transfer (CT) 7](#_Toc138690147)

[**Supplemental figures:** 8](#_Toc138690148)

[Supplemental Figure 1: Sub-group ITT analysis of the primary composite outcome: CCG Trial, western Kenya, 2017-2021 8](#_Toc138690149)

[Supplemental Figure 2: School dropout cox regression estimates by treatment group between mid-study and end of study, per protocol population 9](#_Toc138690150)

[Supplemental Figure 3: Percentage of girls reporting menstrual product use by type and survey round 9](#_Toc138690151)

# **Supplemental methods:**

## Scales

1. A household socio-economic status (SES) absolute index score was constructed as described in Kabudula et al 2017.^1^ Household assets are first weighted by asset type (e.g., household structure floor material) with increasing values for higher SES items. These indicators are categorised into asset types (e.g., dwelling structure, modern assets, power supply, and water and sanitation). Values in each category are normalised into a range of 0-1 with the four subcategories summed to produce on overall SES index score in the range of 0-4. Livestock was not used as one of the asset types due to respondents being adolescents. SES scores was then split into quintiles and dichotomised as ‘poorest’ (quintiles 1-2) and ‘less poor’ (quintiles 3-5).

2. School level water sanitation and hygiene (WASH) scores were compiled with equal weight for (1) water observed for handwashing, (2) soap observed for handwashing; and (3) girl-to-acceptable latrine ratio score. Acceptable latrines were classified as latrines that were clean, without odour, containing a door and roof, with no major holes in the wall, and a stable floor slab. For schools with one or more acceptable latrines: a girl-to-latrine ratio score was built on a distribution wherein schools with 0-30 girls per latrine received 1 point; 30.1-45 girls per latrine received 0.75 points; 45.1-75 points received 0.5 points and 75.1-140 girls per latrine received 0.25points. Schools with ratios over 140-1 latrine got 0 points for this indicator. WASH scores were then dichotomised at the midpoint for the subgroup analysis.

## Sample size re-estimation

The original study was designed to detect a 25% reduction (RR=0.75) in any of the intervention arms with 90% power and an alpha=0.01 to allow for five primary comparisons against the control arm, assuming an ICC of 0.008, based on previous studies.^2-4^ This required 84 schools (21 per arm) assuming an average of 46 girls per school, with 35 girls per school estimated to contribute to the primary analysis after allowance for loss-to-follow and an incidence risk of the primary endpoint of 44.1% in the control school. The sample size was increased to 96 schools (24/arm) following a blinded sample size re-estimation in 2017 due to lower than anticipated enrolment and outcome prevalence in the region (details published elsewhere^5^). The anticipated average number of girls per school was also adjusted from 46 to 41.25, totalling 3,960 schoolgirls, of whom 3,168 were expected to contribute to the analysis after allowing for 20% loss-to-follow-up. The primary comparisons were also changed to 3 instead of 5 (each of the three intervention groups against the control group only). This revised sample size of 96 schools was anticipated to allow the detection of a 25.7% reduction (RR=0.743) in any of the intervention arms with 90% power, an alpha=0.0167, and an ICC of 0.008.

## Other secondary outcomes

Data were also collected for additional secondary outcomes including sexual behaviours (sexual debut, coercive sex, transactional sex, number of sexual partners, age-discordance of partners, pregnancy, condom use, and use of modern contraceptives), school performance indicators (Kenya Certificate of Secondary Education [KCSE] overall points and grade, grade repetition, school transfer and re-enrolment after dropout, and absenteeism), and wellbeing outcomes (PEDSQL-23, EUROQoL-5D-3L, PHQ-9). These outcomes will be reported on in future manuscripts.

# **Deviations from the study protocol:**

The analysis methodology in the trial Statistical Analysis Plan (SAP) differed from the methods defined in the study protocol. Instead of Generalised Estimating Equations (GEE) mentioned in the protocol, multi-level Mixed-Effects Generalised Linear Models were used with random effects for quad and school clusters in order to account for the hierarchical data structure.

# **Supplemental references:**

1. Kabudula CW, Houle B, Collinson MA, Kahn K, Tollman S, Clark S. Assessing changes in household socioeconomic status in rural South Africa, 2001–2013: a distributional analysis using household asset indicators. *Soc Indic Res* 2017; **133**(3): 1047-73.

2. Baird S, Ferreira FHG, Özler B, Woolcock M. Conditional, unconditional and everything in between: a systematic review of the effects of cash transfer programmes on schooling outcomes. *J Dev Effect* 2015; **6**(1): 1-43.

3. Amornkul PN, Vandenhoudt H, Nasokho P, et al. HIV prevalence and associated risk factors among individuals aged 13-34 years in Rural Western Kenya. *PloS One* 2009; **4**(7): e6470.

4. Ghebremichael M, Habtzgi D, Paintsil E. Deciphering the epidemic synergy of herpes simplex virus type 2 (HSV-2) on human immunodeficiency virus type 1 (HIV-1) infection among women in sub-Saharan Africa. *BMC Res Notes* 2012; **5**: 451.

5. Zulaika G, Kwaro D, Nyothach E, et al. Menstrual cups and cash transfer to reduce sexual and reproductive harm and school dropout in adolescent schoolgirls: study protocol of a cluster-randomised controlled trial in western Kenya. *BMC Public Health* 2019; **19**(1): 1317.

# **Supplemental tables:**

## **Supplemental Table 1: Reasons for dropout (n=464; among total 4137 participants enrolled)**

|  | Total | Cups | Cash | Combined | Controls |
| --- | --- | --- | --- | --- | --- |
|  | (N = 4137) | (N = 1059) | (N = 1080) | (N = 1060) | (N = 938) |
| **Primary reason** | n=464 | n=118 | n=133 | n=115 | n=98 |
| Pregnancy | 252 (54.3) | 63 (53.4) | 81 (60.9) | 58 (50.4) | 50 (51.0) |
| Marriage | 68 (14.7) | 23 (19.5) | 14 (10.5) | 14 (12.2) | 17 (17.3) |
| Ran away from home | 42 (9.1) | 12 (10.2) | 10 (7.5) | 14 (12.2) | 6 (6.1) |
| Lack of school fees | 35 (7.5) | 6 (5.1) | 13 (9.8) | 10 (8.7) | 6 (6.1) |
| Poor school performance | 31 (6.7) | 8 (6.8) | 7 (5.3) | 7 (6.1) | 9 (9.2) |
| Family problems | 13 (2.8) | 2 (1.7) | 3 (2.3) | 5 (4.3) | 3 (3.1) |
| Illness | 9 (1.9) | 1 (0.8) | 2 (1.5) | 3 (2.6) | 3 (3.1) |
| Other | 14 (3.0) | 3 (2.5) | 3 (2.3) | 4 (3.5) | 4 (4.1) |

Dropout refers to obtaining the outcome “school dropout.”

## **Supplemental Table 2: Baseline characteristics in the per protocol population, overall and by arm**

|  | **PP Population** | **Not in PP Population** |  |  | **CUP**  **Group** | **CASH**  **Group** | **COMBINED**  **Group** | **CONTROL**  **Group** |  |
| --- | --- | --- | --- | --- | --- | --- | --- | --- | --- |
|  | (N = 3579) | (N = 558) | **p-value** |  | (N = 840) | (N = 949) | (N = 880) | (N = 910) | **p-value** |
| Age, continuous  median (IQR) | 17.17 (16.2-18.0) | 17.36 (16.4-18.1) | **<0.001** |  | 16.93 (16.0-17.7) | 17.30 (16.3-18.0) | 17.28 (16.3-18.1) | 17.18 (16.4-17.9) | **0.0035** |
| Age group <=17 | 1713 (47.9%) | 252 (45.2%) | 0.337 |  | 456 (54.3%) | 433 (45.6%) | 400 (45.5%) | 424 (46.6%) | **0.0315** |
| Socioeconomic status (lowest two quintiles) | 1558 (43.7%) | 200 (36.1%) | **0.015** |  | 322 (38.4%) | 451 (47.7%) | 417 (47.5%) | 368 (40.8%) | **0.0124** |
| Married | 232 (6.6%) | 33 (6.0%) | 0.595 |  | 55 (6.5%) | 62 (6.5%) | 47 (5.3%) | 68 (7.5%) | 0.3557 |
| Orphaned | 106 (3.0%) | 31 (5.6%) | **0.002** |  | 22 (2.6%) | 35 (3.7%) | 26 (3.0%) | 23 (2.5%) | 0.4694 |
| HIV + | 59 (1.7%) | 16 (2.9%) | **0.047** |  | 8 (1.0%) | 16 (1.7%) | 17 (1.9%) | 18 (2.0%) | 0.3464 |
| HSV-2 + | 616 (17.5%) | 85 (15.7%) | 0.343 |  | 133 (16.2%) | 172 (18.5%) | 139 (16.0%) | 172 (19.2%) | 0.3412 |
| Early menarche <13yrs | 212 (5.9%) | 24 (4.3%) | 0.147 |  | 47 (5.6%) | 51 (5.4%) | 56 (6.4%) | 58 (6.4%) | 0.8919 |
| Sexual activity (Yes) | 941 (26.7%) | 183 (33.1%) | **0.009** |  | 219 (26.7%) | 265 (28.2%) | 226 (26.0%) | 231 (25.8%) | 0.7749 |
| Prior pregnancy | 123 (13.1) | 23 (12.6) | 0.451 |  | 23 (10.5%) | 38 (14.3%) | 37 (16.4%) | 25 (10.8%) | 0.2382 |
|  | | | | | | | | | |
| School size >46 | 1807 (50.5%) | 227 (40.7%) | 0.973 |  | 410 (48.8%) | 590 (62.2%) | 426 (48.4%) | 381 (41.9%) | 0.4582 |
| Water sanitation and hygiene school score <1 | 2016 (56.3%) | 335 (60.0%) | 0.992 |  | 396 (47.1%) | 695 (73.2%) | 470 (53.4%) | 455 (50.0%) | 0.6392 |
| Subcounty |  |  | 0.778 |  |  |  |  |  | 0.9999 |
| Gem | 851 (23.8%) | 130 (23.3%) |  |  | 215 (25.6%) | 189 (19.9%) | 265 (30.1%) | 182 (20.0%) |  |
| Rarieda | 802 (22.4%) | 108 (19.4%) |  |  | 158 (18.8%) | 234 (24.7%) | 195 (22.2%) | 215 (23.6%) |  |
| Siaya | 707 (19.8%) | 121 (21.7%) |  |  | 156 (18.6%) | 187 (19.7%) | 147 (16.7%) | 217 (23.8%) |  |
| Ugenya | 665 (18.6%) | 128 (22.9%) |  |  | 181 (21.5%) | 166 (17.5%) | 152 (17.3%) | 166 (18.2%) |  |
| Ugunja | 554 (15.5%) | 71 (12.7%) |  |  | 130 (15.5%) | 173 (18.2%) | 121 (13.8%) | 130 (14.3%) |  |

## **Supplemental Table 3: Per protocol population sensitivity analysis of primary outcome and disaggregated components**

|  | **Cup**  **group** | **Cash group** | **Combined group** | **Control group** | **Unadjusted**  **Cup vs Control** | **Unadjusted**  **Cash vs Control** | **Unadjusted Combined vs Control** | **Adjusted***  **Cup vs Control** | **Adjusted***  **Cash vs Control** | **Adjusted***  **Combined vs Control** |
| --- | --- | --- | --- | --- | --- | --- | --- | --- | --- | --- |
|  | (N = 918)  N (%) | (N = 978)  N (%) | (N = 943)  N (%) | (N = 916)  N (%) | RR (95%CI) p-value | RR (95%CI) p-value | RR (95%CI) p-value | aRR (95%CI) p-value | aRR (95%CI) p-value | aRR (95%CI) p-value |
| Primary outcome of composite HIV, HSV-2, dropout | 179 (19.5) | 220 (22.5) | 223 (23.7) | 180 (19.9) | 1.00 (0.78, 1.27) 0.973 | 1.20 (0.95, 1.52) 0.122 | 1.18 (0.94, 1.49) 0.148 | 1.03 (0.80, 1.32) 0.820 | 1.19 (0.93, 1.52) 0.170 | 1.21 (0.95, 1.54) 0.115 |
| HIV incident infection by end of study | 8 (1.0) | 13 (1.6) | 8 (0.9) | 10 (1.3) | 0.76 (0.56, 2.87) 0.567 | 1.27 (0.56, 2.87) 0.572 | 0.73 (0.29, 1.85) 0.512 | 0.76 (0.30, 1.93) 0.561 | 1.39 (0.60, 3.19) 0.444 | 0.82 (0.32, 2.10) 0.685 |
| HSV-2 incident infection by end of study | 62 (8.8) | 88 (13.0) | 111 (15.0) | 79 (12.1) | 0.66 (0.46, 0.96) 0.028 | 1.02 (0.72, 1.44) 0.907 | 1.18 (0.86, 1.62) 0.309 | 0.76 (0.51, 1.13) 0.173 | 1.06 (0.72, 1.56) 0.768 | 1.25 (0.87, 1.80) 0.220 |
| School dropout by end of study | 113 (12.3) | 127 (13.0) | 111 (11.8) | 97 (10.7) | 1.22 (0.88, 1.68) 0.233 | 1.34 (0.97, 1.84) 0.075 | 1.10 (0.80, 1.52) 0.558 | 1.25 (0.91, 1.71) 0.166 | 1.29 (0.94, 1.76) 0.111 | 1.13 (0.82, 1.55) 0.452 |
| Incident pregnancy (both survey and reported) | 89 (9.9) | 109 (12.1) | 92 (9.9) | 83 (9.6) | 1.04 (0.76, 1.41) 0.825 | 1.30 (0.96, 1.75) 0.092 | 1.04 (0.76, 1.41) 0.825 | 1.05 (0.78, 1.42) 0.746 | 1.23 (0.92, 1.66) 0.163 | 1.01 (0.75, 1.37) 0.932 |

* Includes all dropouts without intervention use data as intervention users

## **Supplemental Table 4: Per protocol population restricted follow-up analysis of primary outcome and disaggregated components**

|  | **Cup**  **group** | **Cash group** | **Combined group** | **Control group** | **Unadjusted**  **Cup vs Control** | **Unadjusted**  **Cash vs Control** | **Unadjusted Combined vs Control** | **Adjusted***  **Cup vs Control** | **Adjusted***  **Cash vs Control** | **Adjusted***  **Combined vs Control** |
| --- | --- | --- | --- | --- | --- | --- | --- | --- | --- | --- |
|  | (N =833)  N (%) | (N =891)  N (%) | (N =870)  N (%) | (N =886)  N (%) | RR (95%CI) p-value | RR (95%CI) p-value | RR (95%CI) p-value | aRR (95%CI) p-value | aRR (95%CI) p-value | aRR (95%CI) p-value |
| Primary outcome of composite HIV, HSV-2, dropout | 101 (16.2) | 189 (30.4) | 158 (25.4) | 174 (28.0) | **0.59 (0.45, 0.78) 0.0002** | 1.10 (0.87, 1.40) 0.4256 | 0.90 (0.71, 1.15) 0.4068 | **0.61 (0.47, 0.80) 0.0002** | 1.09 (0.87, 1.37) 0.4422 | 0.88 (0.70, 1.10) 0.2609 |
| HIV incident infection by end of study | 8 (20.5) | 13 (33.3) | 8 (20.5) | 10 (25.6) | 0.77 (0.31, 1.94) 0.5810 | 1.28 (0.56, 2.90) 0.5577 | 0.74 (0.29, 1.87) 0.5300 | 0.77 (0.30, 1.96) 0.5794 | 1.40 (0.61, 3.22) 0.4323 | 0.83 (0.33, 2.12) 0.7049 |
| HSV-2 incident infection by end of study | 62 (18.5) | 86 (25.6) | 109 (32.4) | 79 (23.5) | **0.67 (0.46, 0.96) 0.0314** | 1.01 (0.72, 1.43) 0.9445 | 1.18 (0.86, 1.62) 0.3151 | 0.76 (0.51, 1.13) 0.1793 | 1.05 (0.72, 1.54) 0.8077 | 1.24 (0.87, 1.78) 0.2318 |
| School dropout by end of study | 35 (12.9) | 98 (36.0) | 48 (17.6) | 91 (33.5) | **0.41 (0.26, 0.64) 0.0001** | 1.11 (0.77, 1.60) 0.5735 | **0.52 (0.34, 0.78) 0.0016** | **0.45 (0.28, 0.71) 0.0008** | 1.08 (0.73, 1.60) 0.7086 | **0.54 (0.35, 0.83) 0.0050** |
| Incident pregnancy (both survey and reported) | 51 (17.5) | 96 (32.9) | 64 (21.9) | 81 (27.7) | **0.66 (0.45, 0.97) 0.0322** | 1.19 (0.85, 1.66) 0.3104 | 0.80 (0.56, 1.14) 0.2221 | **0.69 (0.47, 1.00) 0.0498** | 1.12 (0.81, 1.56) 0.4906 | 0.77 (0.54, 1.10) 0.1559 |

* Includes only follow-up period between mid-study survey and end-study survey

## **Supplemental Table 5: At the margins pooled factorial analysis for the primary outcome**

|  | **CUPS**  (N = 2119) | **NO CUPS**  (N = 2018) | **CASH**  (N = 2140) | **NO CASH**  (N = 1997) | **Unadjusted**  **CUPS VS NO CUPS** | **Unadjusted**  **CASH VS NO CASH** | **INTERACTION** | **Adjusted***  **CUPS VS NO CUPS** | **Adjusted***  **CASH VS NO CASH** | **INTERACTION** |
| --- | --- | --- | --- | --- | --- | --- | --- | --- | --- | --- |
|  | N (%) | N (%) | N (%) | N (%) | RR (95%CI) p-value | RR (95%CI) p-value | p-value | aRR (95%CI) p-value | aRR (95%CI) p-value | p-value |
| ITT Population: Primary outcome of composite HIV, HSV-2, dropout | 424 (20.1) | 419 (20.9) | 470 (22.1) | 373 (18.8) | 0.94 (0.75, 1.19) 0.6074 | 1.14 (0.91, 1.42) 0.2518 | 0.7872 | 0.97 (0.76, 1.24) 0.8232 | 1.14 (0.90, 1.45) 0.2666 | 0.9075 |
| PP Population: Primary outcome of composite HIV, HSV-2, dropout | 261 (15.2) | 365 (19.8) | 351 (19.2) | 275 (15.8) | **0.61 (0.46, 0.80) 0.0003** | 1.06 (0.84, 1.35) 0.6116 | **0.0480** | **0.65 (0.48, 0.88) 0.0058** | 1.07 (0.81, 1.40) 0.6380 | 0.1341 |

*Covariates for adjustment: age, site, school WASH conditions, baseline HIV and HSV-2 status, socioeconomic status, and baseline reported sexual activity. Abbreviations: ITT = intention to treat population; PP = per protocol population; RR = risk ratio; aRR = adjusted risk ratio; statistically significant at p<0.05

## **Supplemental Table 6: Cash use characteristics among girls receiving cash transfer (CT)**

|  | **Mid-study follow-up** | | | **End of study follow -Up*** | | |
| --- | --- | --- | --- | --- | --- | --- |
|  | Total | Cash | Combined | Total | Cash | Combined |
|  | (n=1347) | (n=622) | (n=725) | (n=1536) | (n=753) | (n=783) |
| Spent last CT on the following: | **Select a single primary item** | | | **Select all that apply*** | | |
| Personal clothes/things | 323 (24.0) | 124 (19.9) | 199 (27.4) | 852 (64.2) | 396 (60.2) | 441 (68.2) |
| School clothes | 208 (15.4) | 57 (9.2) | 149 (20.6) | 706 (53.2) | 327 (49.7) | 355 (56.7) |
| Food/drink | 9 (0.7) | 2 (0.3) | 7 (1.0) | 320 (24.1) | 150 (22.5) | 170 (25.8) |
| Soap | 27 (2.0) | 9 (1.4) | 18 (2.5) | 878 (66.1) | 435 (65.4) | 441 (66.8) |
| Book | 219 (16.3) | 60 (9.6) | 159 (21.9) | 928 (69.9) | 429 (64.5) | 485 (75.3) |
| Phone | 2 (0.2) | 0 (0.0) | 2 (0.3) | 31 (2.3) | 18 (2.8) | 12 (1.8) |
| Pads for monthly period | 481 (35.7) | 349 (56.1) | 132 (18.2) | 857 (64.5) | 475 (79.8) | 315 (49.1) |
| Exams/grades | 15 (1.1) | 3 (0.5) | 12 (1.7) | 92 (6.9) | 52 (7.9) | 38 (5.9) |
| House things | 14 (1.0) | 5 (0.8) | 9 (1.2) | 174 (13.1) | 94 (14.1) | 80 (12.1) |
| Favours | 4 (0.3) | 0 (0.0) | 4 (0.6) | 115 (8.7) | 61 (9.1) | 54 (8.2) |
| Other | 47 (3.5) | 13 (2.1) | 34 (4.7) | 112 (8.4) | 52 (8.2) | 53 (8.6) |
| Does anyone ask you for this money? | **Select all that apply** | | | **Select all that apply** | | |
| Father | 131 (9.7) | 59 (9.5) | 72 (9.9) | 138 (9.0) | 73 (9.7) | 65 (8.3) |
| Mother | 461 (34.2) | 220 (35.4) | 241 (33.2) | 581 (26.6) | 300 (39.8) | 281 (35.9) |
| Sibling | 282 (21.1) | 129 (20.7) | 153 (21.1) | 408 (26.6) | 216 (28.7) | 192 (24.5) |
| Other family | 99 (7.3) | 49 (7.9) | 50 (6.9) | 94 (6.1) | 48 (6.4) | 46 (5.9) |
| Friend (girl) | 235 (17.4) | 111 (17.8) | 124 (17.1) | 271 (17.6) | 136 (18.1) | 135 (17.2) |
| Friend (boy) | 29 (2.2) | 14 (2.3) | 15 (2.1) | 36 (2.3) | 17 (2.3) | 19 (2.4) |
| Boyfriend | 15 (1.1) | 6 (1.0) | 9 (1.2) | 21 (1.4) | 7 (0.9) | 14 (1.8) |
| Other | 26 (1.9) | 11 (1.8) | 15 (2.1) | 21 (1.4) | 13 (1.7) | 8 (1.0) |
| Who keeps CT cash card? ‡ |  |  |  |  |  |  |
| Girl |  |  |  | 1116 (82.5) | 521 (79.8) | 595 (85.1) |
| Parent/guardian |  |  |  | 200 (14.8) | 113 (17.3) | 87 (12.4) |

*At F2 girls were allowed to select more than one item. ^208 girls missing F2 data on items purchased (n=590 cash and 606 combined). ^184 girls missing F2 data on who keeps cash card (n=653 cash and 699 combined) ‡Only asked at F2 follow-up.

# **Supplemental figures:**

## ***
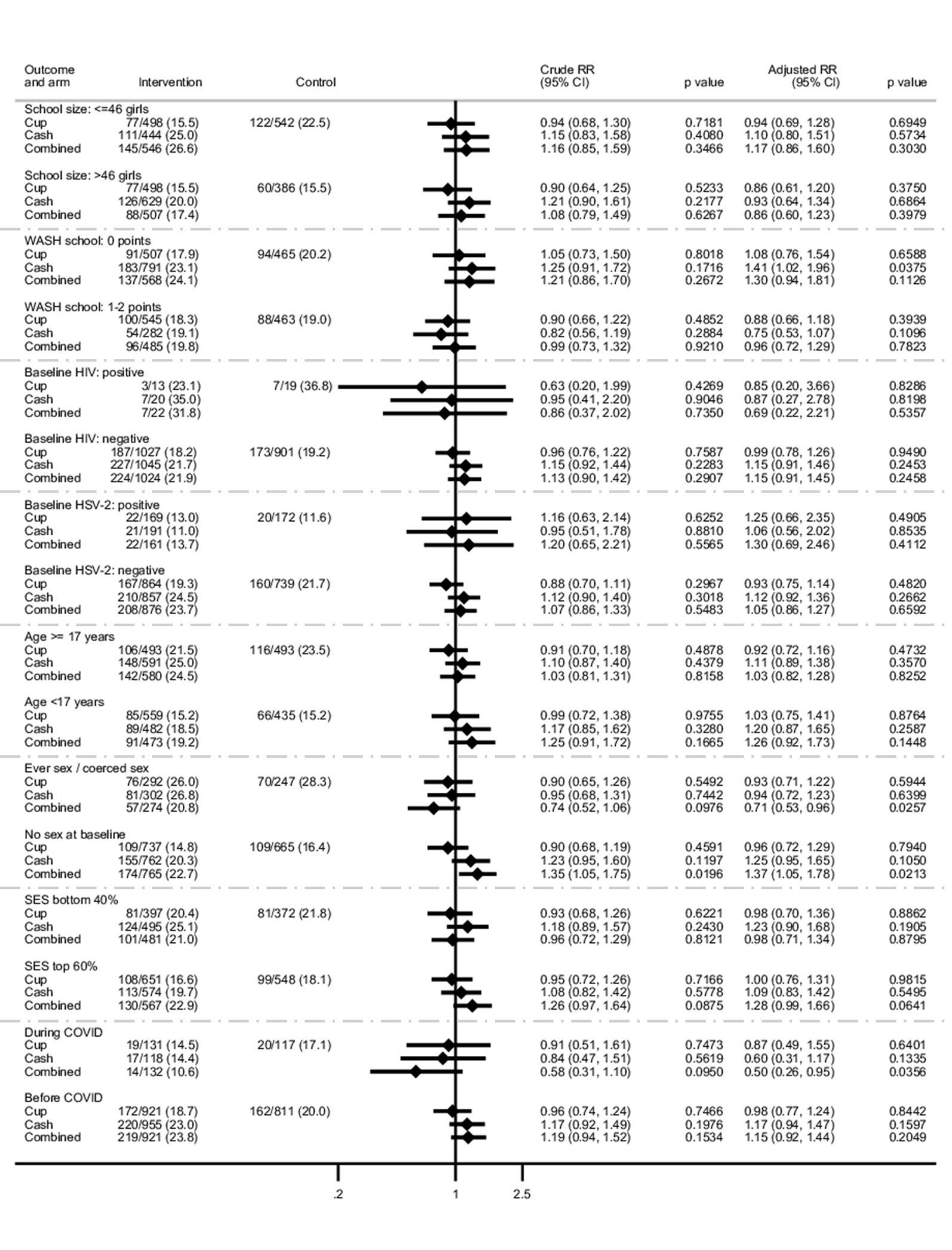
*Supplemental Figure 1: Sub-group ITT analysis of the primary composite outcome: CCG Trial, western Kenya, 2017-2021**

## **Supplemental Figure 2: School dropout cox regression estimates by treatment group between mid-study and end of study, per protocol population**


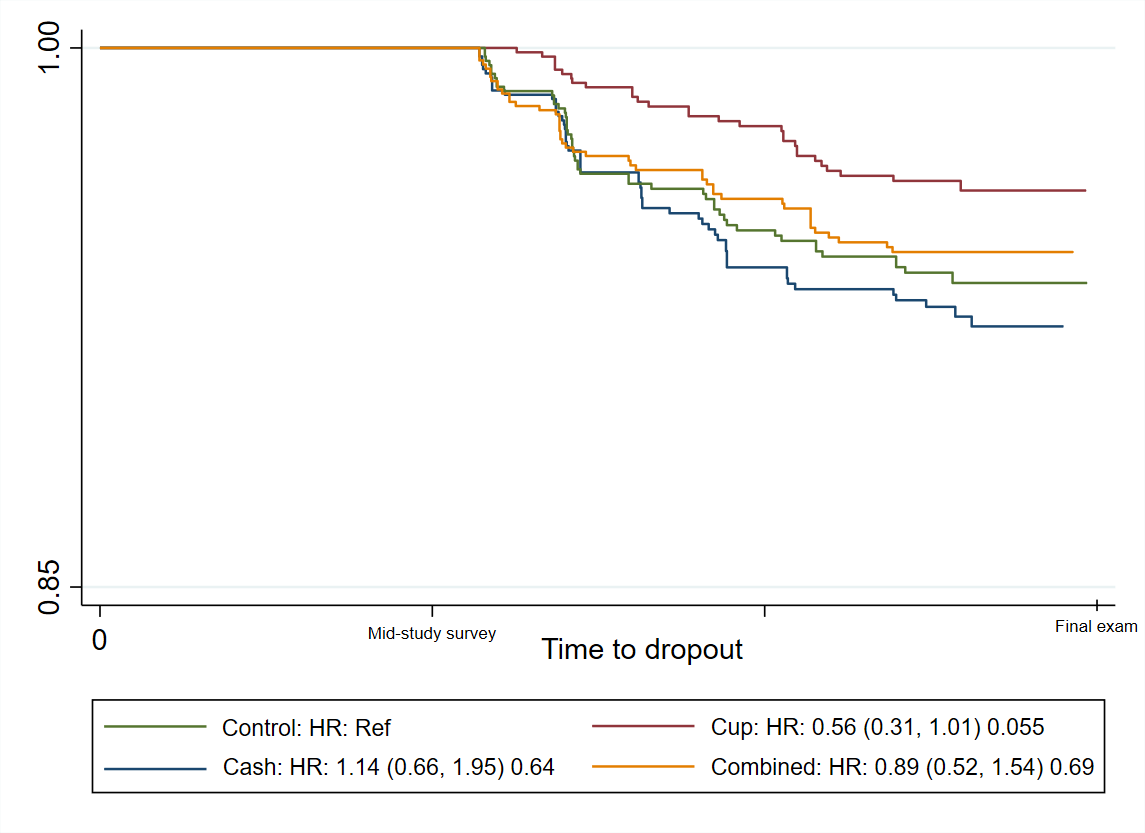


## **
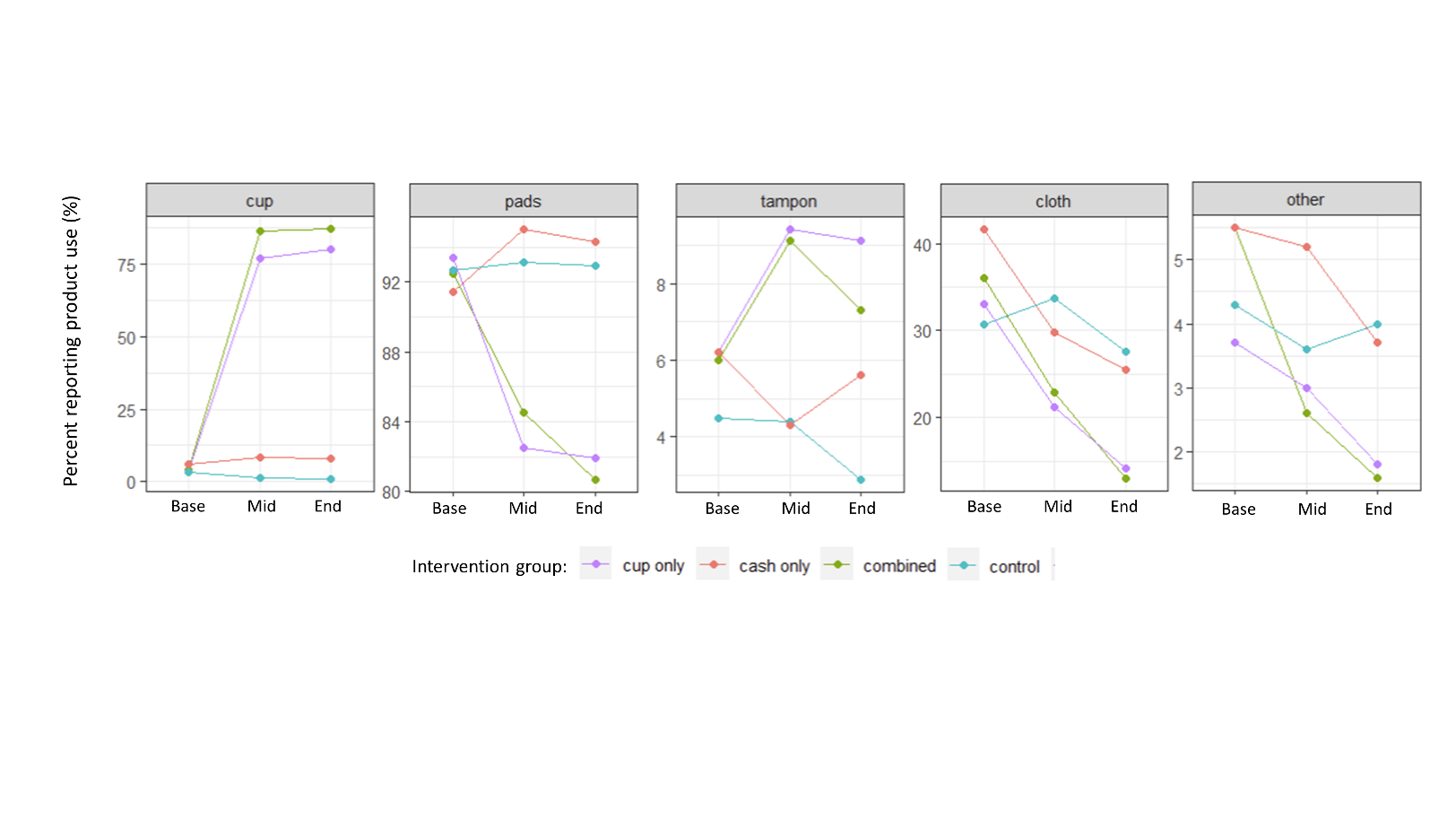
Supplemental Figure 3: Percentage of girls reporting menstrual product use by type and survey round**
